# Longitudinal Associations Among Socioeconomic Status, Delay Discounting, and Substance Use in Adolescence

**DOI:** 10.1007/s10964-024-01989-6

**Published:** 2024-05-03

**Authors:** Kristin M. Peviani, Claudia Clinchard, Warren K. Bickel, Brooks Casas, Jungmeen Kim-Spoon

**Affiliations:** 1 Department of Psychology, Virginia Tech, Blacksburg, VA 24061 USA; 2 Fralin Biomedical Research Institute at VTC, Roanoke, VA 24016 USA

**Keywords:** Socioeconomic status, Delay discounting, Substance use, Growth curve modeling, Adolescence

## Abstract

It is unclear how delay discounting and substance use develop across adolescence and whether contextual factors alter their trajectories. The present study used a longitudinal design to examine whether socioeconomic status is related to developmental trajectories of delay discounting and substance use across adolescence. The sample included 167 adolescents (*M*_age_ = 14 at Time 1; 53% male) and their parents who participated annually across four years. Parents reported SES at Time 1 and adolescents completed delay discounting behavioral assessments and substance use questionnaires at Times 1 to 4. Bivariate latent growth curve modeling revealed that low SES was related to steeper increases in substance use from age 14 through 17, mediated through elevated delay discounting at age 14. The findings clarify the mediating role of delay discounting in linking family economic environment to the progression of substance use.

## Introduction

Extant evidence suggests that a graded association exists between socioeconomic status (SES) and health risk behaviors, including substance use, throughout adulthood, existing from the lowest end of the SES spectrum through the highest end (Bickel et al., 2014 for a review). However, little is known about the role of SES in the development of substance use during adolescence and how the effect of SES on substance use may occur. In the current literature, available cross-national research using data from retrospective cohort studies reports a significant association between lower levels of family socioeconomic status and higher substance use, as well as increased risk of transition to heavy use (Aschengrau et al., 2021; Legleye et al., 2011). The present study used prospective longitudinal data to examine developmental processes relating SES in early adolescence to substance use progression (i.e., increases in the use of substances) throughout adolescence.

Delay discounting, which is the preference for smaller, relatively immediate rewards over larger rewards with a delay, is a common factor of externalizing psychopathology (Castellanos-Ryan et al., 2016). Delay discounting may be an etiological predictor of, and an intermediate phenotype for, substance use disorders (Kwako et al., 2018; MacKillop, 2013; Mitchell, 2019). According to the Competing Neurobehavioral Decision Systems theory, the subcortical regions associated with the impulsive system and the prefrontal regions associated with the executive system work in tandem to influence decision making (Bickel et al., 2007). These competing systems develop asynchronously during adolescence; the impulsive system develops first, whereas executive development is protracted and may continue through the mid-twenties (Casey & Jones, 2010). The theory posits that dysregulation of these competing systems can lead to pathological reinforcement and subsequent substance use disorders, particularly when delay discounting is heightened and harmful reinforcers (e.g., substances) are overvalued (Bickel et al., 2017). From a neurobiological perspective, the maturational imbalance between subcortical regions associated with impulsive behaviors and prefrontal regions associated with executive functioning may leave adolescents vulnerable to heightened substance use (Casey & Jones, 2010). It has been proposed that delay discounting underlies the association between SES and substance use disorders, such that stress and insufficient cognitive resources associated with low SES contribute to heightened delay discounting via restricting the executive system (Bickel et al., 2014). However, no empirical study has examined developmental processes through which SES and delay discounting may contribute to substance use progression.

### Delay Discounting and Substance use Progression during Adolescence

To date, literature presents mixed findings regarding developmental patterns of delay discounting across adolescence. Some research suggests decreases in delay discounting from early adolescence to late adolescence. Specifically, a cross-sectional study examining age differences in delay discounting demonstrated that pre-to-early adolescents (10–13 years) showed significantly higher levels of delay discounting than late adolescents and adults (16–30 years), with middle adolescents (14–15 years) falling in-between (Steinberg et al., 2009). Similarly, a longitudinal study tracking individuals aged 9 to 23 for 10 years found that delay discounting decreases across adolescence, eventually plateauing in late adolescence (Klein et al., 2022). However, other research suggests stable levels of delay discounting across adolescence. For example, a longitudinal study reported that delay discounting was relatively stable between the ages of 15 and 20 (Audrain-McGovern et al., 2009). Likewise, another longitudinal study tracked 13 to 15-year-olds across six years and found that delay discounting was stable across adolescence (Felton et al., 2020).

Regardless of developmental patterns of delay discounting during adolescence, delay discounting has been associated with frequency of substance use, including cigarette, alcohol, and cannabis use (Audrain-McGovern et al., 2009; Felton et al., 2020; Kim-Spoon et al., 2015). Further, preliminary evidence suggests that delay discounting influences substance use, but not the other way around (Audrain-McGovern et al., 2009). However, it is important to note that this study measured delay discounting solely during adolescence, specifically the 10th grade, with two follow-ups during young adulthood (approximately 18–19 and 19–20 years of age). Consequently, developmental trajectories of delay discounting across adolescence could not be examined. Nonetheless, these results indicate that delay discounting predicted trends in smoking progression, whereas smoking did not significantly predict trends in delay discounting. Collectively, although the empirical evidence of the development of delay discounting across adolescence is conflicting, the findings consistently point to high delay discounting as a risk factor for substance use progression.

Additionally, there is some literature suggesting that addressing substance use problems can reduce delay discounting (Lee et al., 2015). However, this reduction in delay discounting at the end of substance use treatment was stronger for adults compared to adolescents, indicating a need for further research on the associations between substance use and delay discounting in adolescents.

### SES Effects on Delay Discounting and Substance use Progression

Although prior conceptual work has linked SES with delay discounting and substance use (Bickel et al., 2014), little is known about the underlying processes through which SES is related to delay discounting and substance use progression. Environments that are unpredictable or resource scarce may impose cognitive strain (Hyde et al., 2020; Mani et al., 2013), thus biasing decision making to favor immediate rewards over long-term gains (Bickel et al., 2014). These effects are posited to be distinct from stress effects, which are also known to be detrimental to executive functioning (Lawson et al., 2018). Such cognitive strain can be explained by a present orientation that favors immediate instead of delayed rewards (Frankenhuis et al., 2016). Specifically, socioeconomic adversity may alter brain functioning associated with reward (e.g., striatum, amygdala, insula, nucleus accumbens) and executive (e.g., dorsolateral prefrontal cortex, parietal cortex) systems, thereby elevating the risk for substance use problems via increased delay discounting (Bickel et al., 2016). For example, one study found that cumulative years of public assistance (from age 5 to 16 years) predicted heightened responsivity in the medial prefrontal cortex during monetary reward anticipation (Romens et al., 2015). The brain regions associated with heightened reward responsivity have also been conceptually linked with cue-induced substance arousal (Ersche et al., 2011; Hanlon et al., 2015). Finally, a study of young adults found evidence that cognitive stress associated with poverty can affect neurocognitive function (e.g., working memory), which can in turn lead to greater delay discounting (Oshri et al., 2019). In light of this literature, empirical investigation is needed to clarify whether, and if so, how resource-scarce environments, consistent with low SES, may contribute to substance use progression by way of affective and cognitive dysregulation, evinced by heightened delay discounting.

## Current Study

The present study sought to clarify associations among SES and delay discounting as well as substance use progression across adolescence, a developmental period known to be sensitive to environmental contexts. The present study used latent growth curve modeling to examine the longitudinal associations among SES, delay discounting, and substance use during adolescence. Specifically, it was hypothesized that lower levels of SES would predict higher levels and greater increases in delay discounting and substance use (Hypothesis 1). Second, it was hypothesized that delay discounting would mediate the link between SES and substance use, such that lower SES would be related to higher levels of delay discounting, which, in turn, would be related to greater increases in substance use (Hypothesis 2). The present study further explored a reciprocal effect of substance use on delay discounting by testing the indirect effects of SES on changes in delay discounting, mediated through substance use.

## Methods

### Participants

The current sample was drawn from a broader longitudinal study of adolescent brain and behavioral development and included 167 adolescents between the ages of 13 and 14 at Time 1 (*M* = 14.07 years of age at Time 1; 53% male) and their parents. The adolescents were between the ages of 15 and 17 at Time 4 (*M* = 17.02, *SD* = 0.55). Families were sampled from a southeastern state in the United States, from small cities and rural towns and counties. A total of 157 adolescents were recruited for their participation in the study at Time 1 and an additional 10 adolescents were recruited for participation at Time 2. Over the course of the study, 16 adolescents withdrew participation for reasons such as: losing contact (*n* = 5), declining participation (*n* = 8), extenuating circumstances (*n* = 2), and moving away (*n* = 1). The number of participants was 157 at Time 1, 148 at Time 2, 146 at Time 3, and 148 at Time 4. Adolescents were excluded from participation for contraindications to magnetic resonance imaging. Despite partially missing data from adolescents who did not participate at each time point, the final sample for the analyses included 167 adolescents by using Full Information Maximum Likelihood (FIML) which allows data from all individuals to be included, regardless of their pattern of missing data. Univariate general linear modeling was used to compare adolescents who participated at all four time points to those who did not participate at all four time points. There were no significant differences between them on demographic variables such as age (*p* = 0.97), income (*p* = 0.39), race (dichotomized as white versus non-white; *p* = 0.38), and gender (*p* = 0.71). Adolescent delay discounting and substance use were assessed annually over four years whereas parents reported their SES at Time 1. At the study’s outset, adolescents predominantly self-identified as White (79%), Black (14%), Asian (2%), or Other (5%). At Time 1, mean annual household income was between $35,000 and $49,999.

### Procedures

Participants were recruited via word of mouth, flyers, and recruitment letters for a study related to adolescent brain development and health. Adolescents provided written assent and their parents provided consent in compliance with the university institutional review board-approved protocol. Parents and adolescents were administered questionnaires and assessments separately by trained researchers at the university offices.

### Measures

#### Socioeconomic Status

Parents reported their annual household income and years of education completed by themselves and their spouses (when applicable) via a paper questionnaire. Total annual household income responses ranged from 1 (none or $0 per month) to 15 ($200,000 or more or $16,667 or more per month). Socioeconomic status composite scores were computed using the mean of standardized annual household income and standardized education using the mean of parents’ and spouses’ education (when applicable). Higher scores were indicative of higher SES.

#### Delay Discounting

Reward-dependent decision making was assessed for adolescents using a computerized delay discounting task (Johnson & Bickel, 2002) across all four time points. Adolescents were given a series of hypothetical monetary decisions on a computer screen in which they made intertemporal choices between an immediate monetary reward and a larger monetary reward with a delay. The amount for the delay was held constant at $100 across four delays: one day, one week, one month, and one year. Individual discounting functions were calculated using hyperbolic *k* values (Mazur, 1987) as an index for discounting rate where *I* is the indifference point, *D* is the time to delay, and *k* is the free parameter where discounting rates decline as delays increase in the formula:

$$I=\frac{1}{1+kD}$$

Nonsystematic discounting was identified and excluded from the analysis for violating the assumption of monotonic decreases in discounting function (Johnson & Bickel, 2008). Delay discounting rates at all four time points were natural log transformed in M*plus* prior to estimating the model. Despite the hypothetical nature of the decisions, consistently similar patterns of discounting have been observed between actual and hypothetical rewards, bolstering evidence of external validity for the task (Johnson & Bickel, 2002).

#### Adolescent Substance use

Adolescents were asked to report the frequency of their substance use (cigarette, alcohol, and cannabis use) across all four time points using items adapted from the Youth Risk Behavior Survey (Kann et al., 2015). Substance use items were preceded by the stem, “Which is the most true for you about using….” for each respective substance. Substance use responses ranged from 1 (never used), to 6 (usually use every day). Substance use composite scores were computed at each time point using an average of responses across all three items. Higher scores were indicative of greater substance use. Scale reliability was acceptable with internal consistency of α = 0.75 at Time 1, α = 0.69 at Time 2, α = 0.61 at Time 3, and α = 0.73 at Time 4.

#### Parental Substance use

Parents were asked to report the frequency of their substance use (cigarette, alcohol, and cannabis use) using the same items that the adolescents answered. Substance use composite scores were computed for the baseline using an average of responses across all three items. Higher scores were indicative of greater substance use. Scale reliability was α = 0.48 at baseline.

### Statistical Analysis

Correlations and descriptive statistics (see Table 1) for all study variables were analyzed using SPSS version 27.0, prior to modeling the growth curve model (GCM) in M*plus* version 8.10 (Muthén & Muthén, 1998–2017). Extreme values in excess of three standard deviations from the mean were winsorized and replaced with nearest, non-extreme values. Structural equation modeling (SEM) was utilized to examine the growth trajectories of delay discounting and substance use (Preacher & Hayes, 2008). To determine whether FIML was acceptable, patterns of missingness on all study variables were examined using Little’s MCAR test (Little, 1988). The resulting pattern resembled a ‘missing completely at random’ pattern (χ^2^ = 191.31, df = 182, *p* = 0.30). Thus, FIML was implemented as it is superior to listwise deletion, pairwise deletion, and similar response pattern imputation by retaining statistical power and producing unbiased estimates and robust standard errors (Enders & Bandalos, 2001). GCM estimates were conducted using maximum likelihood including robust standard errors (MLR) which employs a sandwich estimator to arrive at standard errors robust to nonnormality of observations. Model fit indices were examined using the Root Mean Square Error of Approximation (RMSEA), Comparative Fit Index (CFI), and chi-square (χ^2^). As recommended by Little (2013), acceptable model fit was considered to be RMSEA values less than or equal to 0.08 and CFI values greater than or near 0.90. Chi-square goodness of fit tests were used to assess the best fitting univariate growth trajectories. Modification indices were evaluated and model modifications (e.g., constraining residuals) were implemented as necessary. First, the unconditional univariate GCMs were estimated, and chi-square difference tests were conducted to identify the best-fitting growth trajectories of delay discounting and substance use. Next, these univariate models were combined into a bivariate GCM that incorporated SES (grand mean centered) as a predictor to examine its associations with the growth trajectories of delay discounting and substance use.

**Table 1:** Descriptive Statistics and Correlations for SES, Delay Discounting, and Substance Use, Variables

Variables	*M*	*SD*	Range	*1*	*2*	*3*	*4*	*5*	*6*	*7*	*8*
1. SES	−0.005	0.87	−2.28–2.17								
2. Delay Discounting at Time 1	−4.58	2.74	−10.59–0.88	−0.15							
3. Delay Discounting at Time 2	−4.87	2.49	−9.95 to −0.44	−0.18*	0.57*						
4. Delay Discounting at Time 3	−5.16	2.24	−9.62 to −0.96	−0.15	0.50*	0.65*					
5. Delay Discounting at Time 4	−5.65	2.38	−10.25 to −0.91	−0.22*	0.43*	0.62*	0.68*				
6. Substance Use at Time 1	0.13	0.22	0.00–0.83	−0.20*	0.06	0.05	−0.04	0.07			
7. Substance Use at Time 2	0.21	0.31	0.00–1.19	−0.20*	0.09	0.18*	0.09	0.14	0.72*		
8. Substance Use at Time 3	0.34	0.36	0.00–1.29	−0.14	0.12	0.27*	0.20*	0.24*	0.66*	0.81*	
9. Substance Use at Time 4	0.51	0.44	0.00–1.52	−0.19*	0.19*	0.25*	0.25*	0.33*	0.54*	0.68*	0.81*

For Delay discounting and substance use, log-transformed values are presented. Descriptive statistics and correlations for all raw variables are presented in Supplemental Materials, Table S1. Outliers were winsorized (*n* = 4 for SES*, n* = 2 for delay discounting Time 1, *n* = 2 for delay discounting Time 2, *n* = 3 for delay discounting Time 3, *n* = 2 for delay discounting Time 4; *n* = 4 for substance use T1, *n* = 1 for substance T2, *n* = 3 for substance use T3, *n* = 4 for substance use T4)

*p < 0.05

**Fig. 1:**
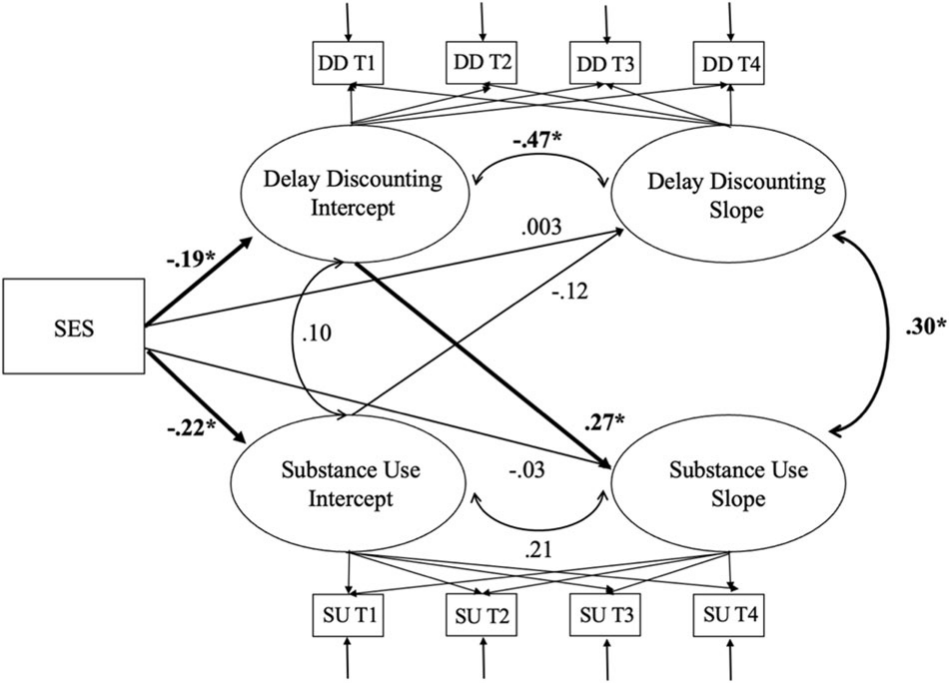
A bivariate growth curve model with SES as a predictor of the growth trajectories of delay discounting and substance use in adolescence. SES socioeconomic status, SU substance use, DD delay discounting, T1 Time ;1, T2 Time 2, T3 Time 3, T4 Time 4. *p < 0.05

To address the first and second hypotheses, SES was included in the bivariate GCM as a predictor (see Fig. 1). This enabled the following: (1) the examination of whether SES was associated with initial status levels and rates of change in delay discounting and substance use and (2) the examination of delay discounting as a mediating process between SES and substance use. In the conditional GCM, within-process slopes and intercepts were correlated. The delay discounting slope was regressed onto the substance use intercept, and the substance use slope was regressed onto the delay discounting intercept. Furthermore, the delay discounting intercept covaried with the substance use intercept and the delay discounting slope covaried with the substance use slope (see Fig. 1). This specification allowed the examination of whether initial levels of SES, as a predictor, predicted the initial levels of delay discounting and substance use and whether the initial level of delay discounting predicted the substance use slope in-turn as well as whether the initial level of substance use predicted the delay discounting slope in-turn. Finally, mediation was tested using the model indirect command in M*plus* with 1000 bootstrap iterations. Maximum Likelihood (ML) estimates of the indirect effects are comparable to those obtained with MLR estimation (Muthén & Muthén, 1998–2017) and were used for conducting bias-corrected bootstrapping tests of indirect effects since bootstrapping is unavailable with MLR estimation (Preacher & Hayes, 2008).

To determine if the effects of delay discounting were separate from the effects of parental modeling and substance use norms on the adolescents’ substance use (Donovan, 2019), each model was tested (i.e., the substance use composite model and individual component models) while controlling for parental substance use. To do so, each model was tested with the intercept and slope of adolescent substance use regressed onto parental substance use, while also correlating SES with parental substance use (see Supplemental Materials for further details).

## Results

Descriptive statistics and correlations for all study variables are presented in Table 1. Substance use composite scores reflect an average of cigarette, alcohol, and cannabis use at each time point. SES composite scores reflect an average of parents’ annual income (*M* = 11.02, *SD* = 2.42) and years of education (*M* = 14.80, *SD* = 2.31) at Time 1. Based on the income-to-needs ratio (as calculated using family income and the number of parents and children in the home), half of the sample was “poor” (25% of the sample had an ITN of less than one) or “near poor” (25% had an ITN of less than two). Outliers were winsorized to retain statistical power and attenuate bias that may occur if eliminated (Ghosh & Vogt, 2012). Skewness and kurtosis of study variables were examined to determine the degree to which the study variables deviated from assumptions of normality (skewness greater than 3 and kurtosis 10; Kline, 1998). All delay discounting variables exhibited skewness in excess of 3 and kurtosis in excess of 10, and one of the substance use variables exhibited skewness greater than 3 (cannabis use at Time 1). Considering this result and the fact that substance use data during younger ages were somewhat positively skewed due to low rates of use, all delay discounting and substance use variables were log-transformed before composites were computed (Kline, 2011).

Multivariate GLM was used to test for the effects of demographic covariates on the study variables. There were no significant effects of sex (*F* = 1.33, *p* = 0.236) or race (*F* = 053, *p* = 0.847; dichotomized as White vs. non-White); thus, covariates were not included in the GCMs. Following the guidelines of Bollen and Curran (2006), the unconditional univariate GCMs were first analyzed for delay discounting and substance use separately using a no growth pattern (intercept only), then a linear growth pattern (by specifying the parameters to 0, 1, and 2), and then a latent growth pattern, which allows the parameters of the slope factor to be estimated freely (fixing the first and second parameters to 0 and 1 for model specification and leaving the third and fourth parameters to be freely estimated). Nested model comparisons were conducted to identify the best fitting growth trajectories using Satorra-Bentler scaled correction factor (Satorra & Bentler, 2001), which is robust to nonnormality compared to alternate difference test statistics (Curran et al., 1996).

Results of the nested model comparisons between the no growth, linear growth, and latent growth univariate models indicated linear growth trajectories for both the delay discounting and substance use models (see Table 2). Thus, all subsequent modeling reflected a linear growth structure for both delay discounting and substance use. In the linear GCM of delay discounting, the significant mean of intercept (*b* = –4.51, *SE* = 0.21, *p* < 0.001) and slope (*b* = –0.31, *SE* = 0.08, *p* < 0.001) indicated that initial delay discounting levels were significantly different from zero and that overall discounting trends declined, respectively. Significant variation was detected in the delay discounting intercept (*b* = 4.57, *SE* = 0.73, *p* < 0.001) as well as the delay discounting slope (*b* = 0.31, *SE* = 0.16, *p* = 0.05), suggesting significant individual differences in delay discounting initial levels and rates of change.

**Table 2:** Chi-Square Difference Test Comparisons of Univariate Delay Discounting and Substance Use Growth Trajectories

Model	χ^2^	*df*	*p*	SB	RMSEA	CFI	Comparison	*T*	Δ*df*	*p*(*d*)
*Delay Discounting*										
a. No Growth	48.30	11	0.000	1.06	0.15	0.71				
b. Linear Growth	**2.53**	**5**	**0.772**	**1.18**	**0.00**	**1.00**	**a vs b**	**50.22**	**6**	**<0.001**
c. Latent Growth	1.80	3	0.616	1.13	0.00	1.00	b vs c	0.76	2	0.685
*Substance Use*										
a. No Growth	316.92	11	0.000	1.34	0.00	0.41				
b. Linear Growth	**8.41**	**5**	**0.135**	**1.07**	**0.06**	**0.99**	**a vs b**	**265.61**	**6**	**<0.001**
c. Latent Growth	4.06	3	0.256	0.93	0.05	1.00	b vs c	3.41	2	0.182

*SB* Satorra-Bentler adjusted chi-square value for model comparison, *CFI* comparative-fit index, *RMSEA* root mean square error of approximation, *T* distributed chi-square with difference in *df*; Δ*df* difference in *df*; *p(d)* probability of the difference tests. Best-fitting models are in bold face

For the linear GCM of substance use, a small negative and non-significant residual variance was constrained to zero to estimate the model. The mean of intercept (*b* = 0.13, *SE* = 0.02, *p* < 0.001) and the mean of slope (*b* = 0.12, *SE* = 0.01, *p* < 0.001) were significant, indicating that initial substance use levels were significantly different from zero and overall substance use trends increased, respectively. Significant variation in substance use intercept (*b* = 0.05, *SE* = 0.01, *p* *<* 0.001) as well as in substance use slope (*b* = 0.01, *SE* = 0.002, *p* < 0.001) were detected, suggesting that there were significant individual differences in substance use initial levels and rates of change.

The conditional bivariate GCM with SES, delay discounting, and substance use demonstrated good model fit (χ^2^ = 34.95, df = 26, *p* = 0.113, RMSEA = 0.05, CFI = 0.98). SES significantly predicted delay discounting and substance use intercepts but not slopes, such that lower SES was associated with higher initial levels of delay discounting and higher initial levels of substance use (see Fig. 1). A significant positive association between delay discounting intercept and substance use slope was observed, indicating that higher initial levels of delay discounting were associated with steeper increases in substance use. Furthermore, delay discounting slope and substance use slope were significantly positively associated, indicating that increasing trends of delay discounting were associated with increasing trends of substance use behaviors. However, substance use intercept did not significantly predict delay discounting slope. Finally, a significant indirect effect was detected from SES at Time 1 to substance use slope via delay discounting intercept, such that lower SES predicted increasing substance use trends via higher initial levels of delay discounting (95% CI [–0.158; –0.00004]). In contrast, the results of testing whether SES predicted delay discounting slope, mediated by the initial levels of substance use, revealed a non-significant indirect effect from SES to delay discounting via substance use (95% CI [–0.018; 0.074]).

In supplemental analyses, the hypothesized model was tested using the individual substances (i.e., cigarette, alcohol, and cannabis) to identify which substance use behaviors may be particularly explained by the effects of SES, mediated through delay discounting (i.e., specificity of the findings). Initial levels of delay discounting did not mediate any of the associations between lower SES and individual substances. Higher initial levels of delay discounting were significantly associated with the progression of cannabis use, which was not seen in the models with cigarette or alcohol use (see Supplemental Materials for further details).

Additionally, to determine whether the effects of delay discounting were separable from the effects of parental substance use on adolescents’ substance use, each of the models was tested (i.e., the substance use composite model and individual substance models) while controlling for parental substance use. While parental substance use was associated with adolescent substance use, the results remained unchanged (see Supplemental Materials for further details).

## Discussion

Understanding associations among SES, delay discounting, and adolescent substance use is important in recognizing how household environments can contribute to individual differences in delay discounting and substance use. The present study used a longitudinal design to examine how environmental contexts, such as SES, may be associated with delay discounting behavior and substance use progression during adolescence, a time when experiences may be especially consequential for brain and behavioral development (Spear, 2013). Specifically, low SES was hypothesized to be associated with elevated initial levels as well as greater increases in both delay discounting and substance use. Delay discounting was hypothesized to mediate the link between SES and developmental changes in substance use. The results indicated that lower SES was associated with higher initial levels, but not changes, of delay discounting and substance use. Elevated initial levels of delay discounting were associated with greater increases in substance use. Importantly, as hypothesized, low SES at baseline was associated with increasing substance use trends via elevated initial levels of delay discounting. The inference about the mediating role of delay discounting in the link between SES and substance use was strengthened by the finding that initial levels of substance use were not significantly associated with inclining trends in delay discounting. This finding is consistent with the literature suggesting that impulsivity may serve as an intermediate phenotype for substance use, rather than merely being a consequence of it (Ersche, et al., 2010).

The current literature presents mixed findings regarding developmental changes of delay discounting across adolescence. In the present study, significant developmental changes in delay discounting were observed, indicating linear decreases across ages 14 to 17. This finding is consistent with prior cross-sectional research suggesting that delay discounting decreases across ages 13 to 20 (Steinberg et al., 2009). This finding is also consistent with a longitudinal study tracking a sample of 9 to 23-year-olds over 10 years, which suggested a linear decrease in delay discounting throughout adolescence that stabilized in late adolescence (Klein et al., 2022). In contrast, longitudinal studies have reported stable delay discounting (i.e., non-significant changes) between 15 and 20 years of age (Audrain-McGovern et al., 2009; Felton et al., 2020). Taken together, the findings appear to indicate that developmental decreases in delay discounting during adolescence can be more sensitively captured when the longitudinal observations span from early to late adolescence.

The Competing Neurobehavioral Decision Systems theory proposes that dysregulation of the reward and executive systems may lead to maladaptive decision making and a greater risk for developing substance use disorders (Bickel et al., 2007). The current finding of the significant linear decrease from age 14 to 17 supports this theoretical perspective by demonstrating that delay discounting was highest during early adolescence, a developmental period when the asynchrony between competing reward and executive systems is putatively imbalanced. Furthermore, not only did the level of delay discounting in early adolescence predict subsequent developmental changes in substance use, but slower decreases in delay discounting were also associated with faster increases in substance use. These findings offer converging behavioral evidence of the Competing Neurobehavioral Decision Systems theory, with respect to the joint development of delay discounting and substance use during adolescence. As such, these results present supporting evidence that delay discounting may serve as an intermediate phenotype for substance use progression (Kwako et al., 2018).

Consistent with the perspective that low SES may heighten developmental decision-making risks by restricting temporal perspective (Bickel et al., 2014), in the present study, low SES was associated with adolescents’ elevated delay discounting, which in turn was associated with an increasing substance use trend. Prior research has implicated future time perspective as a mechanism between uncertain or unpredictable environments and delay discounting (Frankenhuis et al., 2016). As such, clarifying the role of contextual effects on future time perspective and delay discounting development may hold promise for understanding the development of pathological decision making and substance use. Prior research examining the effects of peers on delay discounting suggests social influences increase reward salience (O’Brien et al., 2011). Thus, low SES may impair cognitive and affective regulation to contribute to greater delay discounting (Mani et al., 2013; Mullainathan & Shafir, 2013). Although low SES was concurrently associated with elevated levels of delay discounting and substance use, it was not significantly associated with growth rates of delay discounting or substance use. While income levels may impact delay discounting and substance use in mid-adolescence, there may be other factors at play (i.e., family and school environments often associated with SES, as well as the development of psychopathology that often occurs in adolescence) affecting substance use progression that need to be examined in future work.

The present study examined the impact of SES on delay discounting and substance use during a developmental period when aberrant impulsive decision making and excessive substance use may be indicators of risk for addiction (Magid & Moreland, 2014). The findings from this study may be significant for developing prevention and intervention strategies aimed at reducing economic disparities in substance use. Specifically, demonstrating whether detectable individual differences in delay discounting precede substance use progression may imply that delay discounting serves as an intermediate phenotype for substance use that can be targeted to reduce substance use. Indeed, empirical evidence suggests that delay discounting may be amenable to interventions that encourage the visualization of a future event (Bromberg et al., 2015; Shevorykin et al., 2021). This plasticity may be especially true during adolescence when the development of brain systems involved in delay discounting is ongoing (Bickel et al., 2007). However, further research is needed to examine whether delay discounting can be effectively targeted through interventions aimed at reducing adolescent substance use.

The primary analyses used a composite polysubstance use score computed by averaging the frequency of substance use across three most commonly used substances, (i.e., cigarettes, alcohol, and cannabis). The decision to use the polysubstance use score was based on the literature demonstrating that most youth engage in polysubstance use, such as using both alcohol and cannabis, in contrast to patterns observed among adults who tend to favor one substance (see Halladay et al., 2020 for a review). Further, substance use composites were used given that substance use variables tend to be highly correlated with each other due to the incidence of polysubstance use among adolescents (Conway et al., 2013). Nevertheless, when examined separately (i.e., cigarette, alcohol, and cannabis use), delay discounting did not mediate the link between SES and any individual substances. Given the age range at which substance use was measured in the present sample, the substance use composite may be better at capturing how delay discounting is associated with substance use progression (e.g., also see Felton et al., 2020). Future studies examining later developmental periods (i.e., adulthood) may be able to disentangle any substance use differences associated with delay discounting and SES.

Some limitations to the study should be noted. First, the relatively small sample size and limited time points precluded testing the examination of moderator variables — such as genotype, family history of substance use, and pubertal development—for potentially differential associations among SES, delay discounting, and substance use. Additionally, although the current sample represented the Appalachian region, the sample primarily consisted of White adolescents. Future studies are needed to examine the extent to which the findings can be generalized to other samples, particularly those encompassing greater racial and ethnic diversity, as well as clinical samples. Second, some adolescents began using substances prior to Time 1; thus, it was not possible to confirm whether delay discounting predicted their substance use trends from the time of onset. Future studies using an entirely substance naïve sample to examine the associations between delay discounting and substance use may be useful to clarify the directionality of these associations. Third, the focus on early SES was narrow in order to clarify the predictive role of SES on delay discounting and substance use, but future research should examine SES over a longer time period. Relatedly, although the current study focused on investigating the adverse effects of low SES from an intra-individual approach and its proximal context (i.e., family economic environment), future research examining the SES effects from a broader perspective (e.g., community and societal resources) would offer additional insights into the comprehensive roles of SES in adolescent development. Finally, although the present study demonstrated a significant link between SES and delay discounting, it is unclear whether SES affects the underlying neurobiological processes involved via heightened reward sensitivity, diminished cognitive control, or both. An important direction for future research is to explore associations among SES, neurobiological development of the prefrontal and limbic systems, delay discounting, and substance use to clarify how SES influences both adolescent brain and behavioral development.

## Conclusion

Developmental processes linking socioeconomic status and substance use are not well understood. This study examined longitudinal growth of delay discounting and substance use trajectories across adolescence and examined how socioeconomic risk alters their trajectories. These findings indicate that delay discounting incrementally declines across ages 13 to 17 with higher initial levels of delay discounting posing significant risks for escalating substance use, specifically cannabis use, across adolescence. Significant socioeconomic risks for delay discounting and substance use unfolded across adolescence, indicating that those with low SES faced elevated risks for substance use progression due to heightened delay discounting. As such, these findings highlight the significance of delay discounting in predicting the progression of substance use among adolescents experiencing stress associated with low SES.

## Supplementary Information

Supplemental Materials
