# Supplementary material for: Longitudinal Associations Among Socioeconomic Status, Delay Discounting, and Substance Use in Adolescence
Source: J Youth Adolesc. 2024 May 3;53(12):2706–16. doi: 10.1007/s10964-024-01989-6 (PMC11534883; doi:10.1007/s10964-024-01989-6)
Supplement: Supplementary file 1 — Supplemental Materials [file 10964_2024_1989_MOESM1_ESM.docx]

**Supplemental Materials**

Longitudinal Associations Among Socioeconomic Status, Delay Discounting, and

Substance Use in Adolescence

Kristin M. Peviani, Claudia Clinchard, Warren K. Bickel, Brooks Casas, &

Jungmeen Kim-Spoon

**Results of Individual Substance Use Components**

Descriptive statistics and correlations for all variables are presented in Table S2. We used multivariate GLM to test for the effects of demographic covariates on the study variables for each individual substance use model and found no significant effects of sex (*F* = 1.15 to 1.70; *p* = .103 to .343) or race (*F* = 0.57 to 1.12; *p* = .362 to .818); thus, covariates were not included in the GCMs. We first analyzed the unconditional univariate GCMs for each of the substances and conducted nested model comparisons to identify the best-fitting growth trajectories using the Satorra-Bentler scaled correction factor (Satorra & Bentler, 2001), as was done in the substance use composite and delay discounting models.

**Cigarette Use**

Results of the nested model comparisons between the no growth, linear growth, and latent growth univariate models indicated linear growth trajectories (see Table S3). In the linear GCM of cigarette use, the significant mean of intercept (*b* = 0.09, *SE* = .02, *p* < .001) and slope (*b* = .07, *SE* = .01, *p* < .001) indicated that initial cigarette use levels were significantly different from zero and that overall cigarette use trends increased, respectively. Significant variation was detected in the cigarette use intercept (*b* = 0.04, *SE* = .01, *p* < .001) as well as the cigarette use slope (*b* = .01, *SE* = .003, *p* < .001), suggesting that there were significant individual differences in cigarette use initial levels and rates of change.

The conditional bivariate GCM with SES, delay discounting, and cigarette use demonstrated good model fit (χ^2^ = 29.51, *df* = 26, *p* = .288, RMSEA = .03, CFI = .99). SES significantly predicted delay discounting (*b* = -0.47, *SE* = .24, *p* = .047) and cigarette use intercepts (*b* = -0.05, *SE* = .02, *p* = .007) but not slopes (*b* = 0.02, *SE* = .09, *p* = .874 for delay discounting and *b* = -0.02, *SE* = .01, *p* = .070 for cigarette use). The results suggested that lower SES was associated with higher initial levels of delay discounting and cigarette use. There were no other significant associations observed.

**Alcohol Use**

Results of the nested model comparisons between the no growth, linear growth, and latent growth univariate models indicated linear growth trajectories (see Table S3). The residuals at Times 2 and 3 were correlated, as indicated by modification indices. In the linear GCM of alcohol use, the significant mean of intercept (*b* = 0.24, *SE* = .03, *p* < .001) and slope (*b* = .16, *SE* = .01, *p* < .001) indicated that initial alcohol use levels were significantly different from zero and that overall alcohol use trends increased, respectively. Significant variation was detected in the alcohol use intercept (*b* = 0.11, *SE* = .02, *p* < .001) as well as the alcohol use slope (*b* = .02, *SE* = .004, *p* < .001), suggesting that there were significant individual differences in alcohol use initial levels and rates of change.

The conditional bivariate GCM with SES, delay discounting, and alcohol use demonstrated good model fit (χ^2^ = 40.11, *df* = 26, *p* = .038, RMSEA = .06, CFI = .97). SES significantly predicted delay discounting (*b* = -0.47, *SE* = .24, *p* = .045) and alcohol use intercepts (*b* = -0.07, *SE* = .03, *p* = .034) but not slopes (*b* = -0.01, *SE* = .09, *p* = .941 for delay discounting and *b* = 0.03, *SE* = .02, *p* = .122 for alcohol use). The results indicated that lower SES was associated with higher initial levels of delay discounting and alcohol use. A significant positive association between the delay discounting slope and alcohol use slope was observed (*b* = 0.03, *SE* = .01, *p* = .022), indicating that increases in delay discounting were associated with increases in alcohol use. There were no other significant associations observed.

**Cannabis Use**

Results of the nested model comparisons between the no growth, linear growth, and latent growth univariate models indicated latent growth trajectories (see Table S3). The residuals at Times 2 and 3 were correlated, as indicated by modification indices, and a small negative and non-significant residual variance at Time 1 was constrained to zero to estimate the model. In the latent basis GCM of cannabis use, the significant mean of intercept (*b* = 0.05, *SE* = .01, *p* < .001) and slope (*b* = .44, *SE* = .05, *p* < .001) indicated that initial cannabis use levels were significantly different from zero and that overall cannabis use trends increased, respectively. Significant variation was detected in the cannabis use intercept (*b* = 0.03, *SE* = .01, *p* < .001) as well as the cannabis use slope (*b* = .34, *SE* = .05, *p* < .001), suggesting that there were significant individual differences in cannabis use initial levels and rates of change.

The conditional bivariate GCM with SES, delay discounting, and cannabis use demonstrated good model fit (χ^2^ = 56.11, *df* = 25, *p* < .001, RMSEA = .09, CFI = .93). SES significantly predicted delay discounting (*b* = -0.47, *SE* = .24, *p* = .047) and cannabis use intercepts (*b* = -0.04, *SE* = .02, *p* = .005) but not slopes (*b* = 0.01, *SE* = .09, *p* = .891 for delay discounting and *b* = -0.03, *SE* = .05, *p* = .650 for cigarette use). The results suggested that lower SES was associated with higher initial levels of delay discounting and cannabis use. A significant positive association between the delay discounting intercept and the cannabis use slope was observed (*b* = 0.07, *SE* = .03, *p* = .007), indicating that higher initial levels of delay discounting were associated with steeper increases in cannabis use. However, the indirect effect of SES and cannabis use mediated through delay discounting intercept mediating was not significant (95% CI [-.095; .001]).

**Sensitivity Analyses Controlling for Parental Substance Use**

**Substance Use Composite**

The conditional bivariate GCM with SES, delay discounting, and substance use controlling for parental substance use demonstrated good model fit (χ^2^ = 42.23, *df* = 32, *p* = .107, RMSEA = .04, CFI = .98). SES remained a significant predictor of delay discounting (*b* = -0.47, *SE* = .24, *p* = .047) and substance use intercepts (*b* = -0.05, *SE* = .02, *p* = .007) but not slopes (*b* = -0.002, *SE* = .09, *p* = .981 for delay discounting and *b* = 0.01, *SE* = .01, *p* = .515 for substance use). The significant positive association between the delay discounting intercept and substance use slope remained (*b* = 0.02, *SE* = .01, *p* = .004). The positive association between the delay discounting slope and substance use slope remained (*b* = 0.02, *SE* = .01, *p* = .039). The significant indirect effect from SES to substance use slope via the delay discounting intercept remained (95% CI [-.160; -.0001]). SES was significantly associated with parental substance use (*b* = -0.19, *SE* = .06, *p* = .002), such that higher SES was associated with less parental substance use. Parental substance use predicted significantly the slope of adolescent substance use (*b* = 0.04, *SE* = .01, *p* < .001) but did not significantly predict the intercept (*b* = 0.01, *SE* = .02, *p* = .561) of adolescent substance use.

**Cigarette Use**

The conditional bivariate GCM with SES, delay discounting, and cigarette use controlling for parental cigarette use demonstrated good model fit (χ^2^ = 38.06, *df* = 32, *p* = .213 RMSEA = .03, CFI = .98). SES remained a significant predictor of delay discounting (*b* = -0.47, *SE* = .24, *p* = .047) and cigarette use intercepts (*b* = -0.05, *SE* = .02, *p* = .008) but not slopes (*b* = 0.01, *SE* = .09, *p* = .875 for delay discounting and *b* = -0.02, *SE* = .01, *p* = .073 for cigarette use).. SES was significantly associated with parental cigarette use (*b* = -0.52, *SE* = .12, *p* < .001), such that higher SES was associated with less parental cigarette use. Parental cigarette use did not significantly predict the intercept (*b* = 0.00, *SE* = .01, *p* = .979) or slope (*b* = 0.00, *SE* = .01, *p* = .969) of adolescent cigarette use.

**Alcohol Use**

The conditional bivariate GCM with SES, delay discounting, and alcohol use controlling for parental alcohol use demonstrated good model fit (χ^2^ = 41.38, *df* = 32, *p* = .124, RMSEA = .04, CFI = .98). SES remained a significant predictor of delay discounting (*b* = -0.47, *SE* = .24, *p* = .045) and alcohol use intercepts (*b* = -0.07, *SE* = .03, *p* = .033) but not slopes (*b* = -0.01, *SE* = .09, *p* = .920 for delay discounting and *b* = 0.02, *SE* = .02, *p* = .182 for alcohol use). The significant positive association between the delay discounting slope and alcohol use slope remained (*b* = 0.02, *SE* = .01, *p* = .022).

SES was not significantly associated with parental alcohol use (*b* = .12, *SE* = .06, *p* = .052). Parental alcohol use predicted significantly the slope of adolescent alcohol use (*b* = 0.03, *SE* = .02, *p* = .037), but it did not significantly predict the intercept (*b* = 0.01, *SE* = .03, *p* = .751) of adolescent alcohol use.

**Cannabis Use**

The conditional bivariate GCM with SES, delay discounting, and cannabis use controlling for parental cannabis use demonstrated good model fit (χ^2^ = 63.61, *df* = 31, *p* < .001, RMSEA = .08, CFI = .93). SES remained a significant predictor of delay discounting (*b* = -0.47, *SE* = .24, *p* = .047) and cannabis use intercepts (*b* = -0.04, *SE* = .01, *p* = .006) but not slopes (*b* = 0.01, *SE* = .09, *p* = .895 for delay discounting and *b* = -0.004, *SE* = .05, *p* = .934 for cannabis use). The significant positive association between the delay discounting intercept and the cannabis use slope remained (*b* = 0.07, *SE* = .02, *p* = .004). SES was significantly associated with parental alcohol use (*b* = -0.16, *SE* = .07, *p* = .032), such that higher SES was associated with less parental cannabis use. Parental cannabis use significantly predicted the intercept (*b* = 0.03, *SE* = .02, *p* = .036) and slope (*b* = 0.11, *SE* = .04, *p* = .002) of adolescent cannabis use, indicating that greater parental cannabis use was associated with both higher initial levels and greater increases in adolescent cannabis use.

**Table S1**

*Descriptive Statistics and Correlations of Raw Delay Discounting and Substance Use Variables*

| Variables | *M* | *SD* | *Range* | *1* | *2* | *3* | *4* | *5* | *6* | *7* |
| --- | --- | --- | --- | --- | --- | --- | --- | --- | --- | --- |
| 1. Delay Discounting at Time 1 | 0.16 | 0.46 | 0.00 – 2.42 |  |  |  |  |  |  |  |
| 1. Delay Discounting at Time 2 | 0.06 | 0.12 | 0.00 – 0.65 | .10 |  |  |  |  |  |  |
| 1. Delay Discounting at Time 3 | 0.04 | 0.07 | 0.00 – 0.38 | .23* | .66* |  |  |  |  |  |
| 1. Delay Discounting at Time 4 | 0.03 | 0.08 | 0.00 – 0.40 | .24* | .59* | .43* |  |  |  |  |
| 1. Substance Use at Time 1 | 1.20 | 0.33 | 1.00 – 2.33 | .01 | .08 | .05 | .13 |  |  |  |
| 1. Substance Use at Time 2 | 1.36 | 0.55 | 1.00 – 3.33 | -.03 | .18* | .10 | .22* | .71* |  |  |
| 1. Substance Use at Time 3 | 1.61 | 0.70 | 1.00 – 3.67 | -.04 | .21* | .12 | .18* | .64* | .78* |  |
| 1. Substance Use at Time 4 | 1.97 | 0.94 | 1.00 – 4.67 | .11 | .23* | .18* | .17 | .52* | .64* | .80* |

*Note.* The values presented are the raw, non-log-transformed values.

**p* < .05.

**Table S2, Part 1**

*Descriptives Statistics and Correlations of SES, Delay Discounting, Cigarette, Alcohol, and Cannabis Use*

|  | *Mean* | *SD* | *Range* | 1 | 2 | 3 | 4 | 5 | 6 | 7 | 8 |
| --- | --- | --- | --- | --- | --- | --- | --- | --- | --- | --- | --- |
| 1. SES | -0.005 | 0.87 | -2.28 – 2.17 |  |  |  |  |  |  |  |  |
| 1. DD T1 | -4.58 | 2.74 | -10.59 – 0.88 | -.15 |  |  |  |  |  |  |  |
| 3. DD T2 | -4.87 | 2.49 | -9.95 – -0.44 | -.18* | .57* |  |  |  |  |  |  |
| 4. DD T3 | -5.16 | 2.24 | -9.62 – -0.96 | -.15 | .50* | .65* |  |  |  |  |  |
| 5. DD T4 | -5.65 | 2.38 | -10.25 – -0.91 | -.22* | .43* | .62* | .68* |  |  |  |  |
| 6. Cigarette T1 | 0.09 | 0.24 | 0.00 – 0.69 | -.18* | .02 | .04 | .00 | .03 |  |  |  |
| 7. Cigarette T2 | 0.17 | 0.34 | 0.00 – 1.10 | -.20* | .03 | .11 | -.01 | .05 | .55* |  |  |
| 8. Cigarette T3 | 0.21 | 0.37 | 0.00 – 1.10 | -.26* | .01 | .14 | .03 | .14 | .58* | .72* |  |
| 9. Cigarette T4 | 0.32 | 0.47 | 0.00 – 1.39 | -.21* | .13 | .17 | .13 | .21* | .40* | .53* | .75* |

**Table S2, Part 2**

*Descriptives Statistics and Correlations of SES, Delay Discounting, Cigarette, Alcohol, and Cannabis Use*

|  | *Mean* | *SD* | *Range* | 9 | 10 | 11 | 12 | 13 | 14 | 15 |
| --- | --- | --- | --- | --- | --- | --- | --- | --- | --- | --- |
| 9. Alcohol T1 | 0.25 | 0.37 | 0.00 – 1.10 |  |  |  |  |  |  |  |
| 10. Alcohol T2 | 0.34 | 0.47 | 0.00 – 1.39 | .58* |  |  |  |  |  |  |
| 11. Alcohol T3 | 0.51 | 0.53 | 0.00 – 1.39 | .51* | .68* |  |  |  |  |  |
| 12. Alcohol T4 | 0.72 | 0.52 | 0.00 – 1.39 | .43* | .58* | .73* |  |  |  |  |
| 13. Cannabis T1 | 0.05 | 0.18 | 0.00 – 0.69 | .46* | .16 | .20* | .14 |  |  |  |
| 14. Cannabis T2 | 0.13 | 0.34 | 0.00 – 1.10 | .48* | .38* | .28* | .18* | .63* |  |  |
| 15. Cannabis T3 | 0.30 | 0.53 | 0.00 – 1.61 | .47* | .49* | .45* | .34* | .40* | .71* |  |
| 16. Cannabis T4 | 0.48 | 0.64 | 0.00 – 1.79 | .47* | .51* | .48* | .48* | .38* | .52* | .73* |

**Table S2, Part 3**

*Descriptives Statistics and Correlations of SES, Delay Discounting, Cigarette, Alcohol, and Cannabis Use*

|  | 1. SES | 2. DD T1 | 3. DD T2 | 4. DD T3 | 5. DD T4 | 6. Cig T1 | 7. Cig T2 | 8. Cig T3 | 9. Cig T4 |
| --- | --- | --- | --- | --- | --- | --- | --- | --- | --- |
| 9. Alcohol T1 | -.13 | .13 | .06 | -.03 | .14 | .44* | .54* | .41* | .45* |
| 10. Alcohol T2 | -.15 | .19* | .17* | .14 | .21* | .21* | .47* | .34* | .37* |
| 11. Alcohol T3 | -.001 | .16 | .20* | .23* | .22* | .21* | .32* | .23* | .29* |
| 12. Alcohol T4 | -.02 | .20* | .09 | .23* | .28* | .16 | .30* | .28* | .41* |
| 13. Cannabis T1 | -.21* | -.06 | .01 | -.06 | -.06 | .59* | .47* | .44* | .34* |
| 14. Cannabis T2 | -.14 | -.04 | .16 | .06 | .06 | .47* | .56* | .42* | .43* |
| 15. Cannabis T3 | -.11 | .07 | .27* | .16 | .18* | .39* | .49* | .36* | .42* |
| 16. Cannabis T4 | -.25* | .14 | .30* | .21* | .28* | .29* | .48* | .43* | .56* |

*Note.* SES*= socioeconomic status;* DD = Delay Discounting; Cig = Cigarettes; T1 = Time 1; T2 = Time 2; T3 = Time 3; T4 = Time 4. For Delay discounting, cigarette use, alcohol use, and cannabis use, log-transformed values are presented. Outliers were winsorized (*n* = 4 for SES*, n* = 2 for delay discounting T1, *n* = 2 for delay discounting T2, *n* = 3 for delay discounting T3, *n* = 2 for delay discounting T4; *n* = 4 for cigarettes T1, *n* = 1 for cigarettes T2, *n* = 3 for cigarettes T3, *n* = 4 for cigarettes T4, *n* = 3 for alcohol T1, *n* = 9 for cannabis T1, *n* = 7 for cannabis T2, *n* = 2 for cannabis T3).

| **Table S3**  *Chi-Square Difference Test Comparisons of Univariate Individual Substance Use Growth Trajectories* | | | | | | | | | | | | | |
| --- | --- | --- | --- | --- | --- | --- | --- | --- | --- | --- | --- | --- | --- |
| Model | χ^2^ | *df* | *p* | SB | RMSEA | CFI | Comparison | *T* | Δ*df* | | | *p*(*d*) | |
|  | | | | | | | | | | | | | |
| *Cigarette Use* | | | | | | | | | | | | | |
| a. No Growth | 135.65 | 11 | .000 | 1.52 | .26 | .00 |  |  |  | | |  | |
| **b. Linear Growth** | **9.23** | **5** | **.100** | **1.13** | **.07** | **.97** | **a vs b** | **105.10** | **6** | | | **<.001** | |
| c. Latent Growth | 12.39 | 3 | .006 | 0.66 | .14 | .92 | b vs c | 1.23 | 2 | | | .541 | |
| *Alcohol Use* | | | | | | | | | | | | | |
| 1. No Growth | 250.98 | 11 | .000 | 0.91 | .36 | .00 |  |  | | |  | | |
| 1. **Linear Growth** | **6.38** | **4** | **.172** | **0.90** | **.06** | **.99** | **a vs b** | **243.14** | | **7** | | | **<.001** |
| c. Latent Growth | 2.70 | 2 | .260 | 0.61 | .05 | 1.00 | b vs c | 3.44 | | 2 | | | .179 |
| *Cannabis Use* | | | | | | | | | | | | | |
| 1. No Growth | 289.35 | 11 | .000 | 1.68 | .26 | .00 |  |  | | |  | | |
| 1. Linear Growth | 29.98 | 6 | .000 | 1.37 | .15 | .86 | a vs b | 216.88 | | 5 | | | <.001 |
| **c. Latent Growth** | **3.59** | **3** | **.309** | **.951** | **.03** | **1.00** | **b vs c** | **21.05** | | **3** | | | **<.001** |
| 1. *Note.* SB = Satorra-Bentler adjusted chi-square value; CFI = comparative-fit index; RMSEA = root mean square error of approximation; *T* = distributed chi-square with difference in *df*; Δ*df* = difference in *df*; *p(d)* = probability of the difference tests. Best-fitting models are in bold face. | | | | | | | | | | | | | |

**Reference**

Satorra, A., & Bentler, P. M. (2001). A scaled difference chi-square test statistic for moment structure analysis. *Psychometrika, 66*, 507-514. https://doi.org/10.1007/BF02296192
